# Chelation-Induced Zwitterion-like Antifouling Behavior on Anionic Poly(3,4-ethylenedioxythiophene) Surfaces

**DOI:** 10.1021/acs.langmuir.4c03275

**Published:** 2024-10-09

**Authors:** Tzu-Yu Kao, Ya-Chen Gong, Cheng-Hsun Huang, Yen-Ku Wu, Shyh-Chyang Luo

**Affiliations:** † Department of Materials Science and Engineering, National Taiwan University, No. 1, Sec. 4, Roosevelt Road, Taipei 10617, Taiwan; ‡ Department of Applied Chemistry, National Yang Ming Chiao Tung University, 1001 University Road, Hsinchu 30010, Taiwan

## Abstract

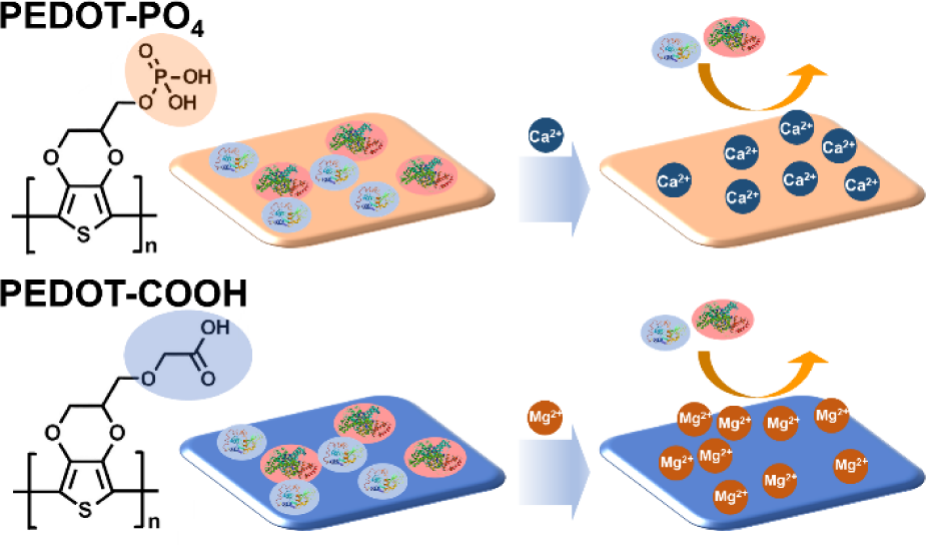

Antifouling properties are crucial for enhancing the longevity and functionality of biomedical implants, drug delivery systems, and biosensors. Zwitterionic polymers are renowned for their exceptional surface hydration and charge neutrality, which effectively resist biomolecular adsorption and protein attachment. We propose an innovative approach to develop zwitterion-like antifouling surfaces by chelating divalent cations with anionic poly(3,4-ethylenedioxythiophene) (PEDOT) films, specifically PEDOT–PO_4_ and PEDOT–COOH. The chelation behavior of these films was systematically evaluated using Na^+^, Mg^2+^, and Ca^2+^ ions. Divalent ions, particularly Ca^2+^ and Mg^2+^, exhibit a strong affinity for the anionic groups, leading to significant antifouling properties. These modified surfaces effectively repelled both negatively charged bovine serum albumin (BSA) and positively charged lysozyme (LYZ) proteins across various pH environments. This study offers valuable insights into the antifouling characteristics of charged surfaces, enhancing our understanding of how ion-mediated surface modifications influence protein adsorption and interactions.

## 1 Introduction

In recent times, antifouling properties have been vital for various widely used materials, as they effectively prevent the accumulation of biological organisms, thereby enhancing the operational longevity of devices and materials. These properties are especially critical in biomedical coatings,^1,2^ drug delivery systems,^3−5^ biosensors,^6^ and membranes.^7,8^ Researches have shown that zwitterionic polymers and polymers containing poly(ethylene glycol) moieties exhibit excellent antifouling activities. This effectiveness is attributed to the strong surface hydration of these polymers, which prevents biomolecules, such as proteins, from displacing the tightly bound surface water molecules and adhering to the surfaces.^9,10^ Additionally, electrostatic interactions are crucial in biofouling, making surface charge a critical factor in maintaining nonfouling properties.^11^ Zwitterionic polymers, which possess an equal balance of positive and negative charges, are particularly effective in this regard.^12^ By keeping electric neutrality, zwitterionic polymers can resist unwanted adsorption^13^ and maintain stability under physiological conditions.^14^

Zwitterionic polymers typically feature quaternary ammonium as a common cationic group and phosphate, carboxylate, or sulfonate as typical anionic groups.^15^ By the design of monomers that feature both cationic and anionic groups on their side chains, a variety of zwitterionic polymers can be synthesized. Some of the most common examples include poly(2-methacryloyloxyethyl phosphorylcholine) (PMPC)^16,17^ and poly(sulfobetaine methacrylate) (PSBMA).^18^ Beyond conventional zwitterionic polymers, recent research has introduced innovative methods for designing zwitterionic materials. Some studies have focused on synthesizing anionic and cationic monomers separately and adjusting their feed ratios to manipulate copolymer surface charge, resulting in unique antifouling properties.^19,20^ Other research has investigated how varying the distance between positive and negative charges within zwitterionic groups affects antifouling effectiveness.^21−23^ Additionally, integrating these antifouling moieties into conducting polymers, such as poly(3,4-ethylenedioxythiophene) (PEDOT),^24^ and immobilizing them onto electrode surfaces has proven effective in preventing nonspecific adsorption^25−27^ and significantly enhancing the sensitivity of biosensors.^28−30^

In addition to controlling surface charge through polymer functional groups, ion chelation can also be used to manipulate surface charge,^31−33^ which raises a compelling question: could we synthesize a polymer with negatively charged groups, such as phosphate and carboxylic groups, and then chelate it with positively charged ions to achieve zwitterion-like antifouling properties by adjusting the ion concentration? To prove this idea, we synthesized two novel negatively charged EDOT monomers: EDOT–PO_4_ (phosphate group) and EDOT–COOH (carboxylic acid group), following a previous study.^34^ Films of PEDOT–PO_4_ and PEDOT–COOH were made via electropolymerization, and their chelation with Na^+^, Mg^2+^, and Ca^2+^ was tested. Divalent ions chelated effectively, with Ca^2+^ showing a higher affinity for PEDOT–PO_4_ and Mg^2+^ for PEDOT–COOH.

Following the identification of ions that chelate effectively to the surfaces, we conducted protein binding tests on cation-chelated anionic PEDOT surfaces. Bovine serum albumin (BSA) and lysozyme (LYZ) were selected for protein adsorption tests. The net charge of a protein is determined by its isoelectric point (pI). BSA, with a pI of approximately 4.9, carries a net negative charge in an aqueous solution at pH 7.0, whereas LYZ, with a pI of around 10.7, carries a net positive charge under the same conditions. By comparing the adsorption behavior of both BSA and LYZ on PEDOT–PO_4_ and PEDOT–COOH surfaces with varying chelation ion concentrations, we aim to elucidate the impact of chelation ions on the interactions between proteins and polymers. Moreover, by manipulating the surface charges, we seek to explore the potential of creating zwitterion-like antifouling surfaces.

## 2 Experimental Section

### 2.1 Materials

Calcium chloride, magnesium chloride, methyl bromoacetate, sodium chloride, sodium hydroxide, sodium hydride, tetrahydrofuran, and sulfuric acid were purchased from Sigma-Aldrich. Hydrogen peroxide was purchased from Honeywell. Tetrabutylammonium perchlorate and dioctyl sodium sulfosuccinate were purchased from Acros. Sodium iodide, lithium perchlorate, and anhydrous acetonitrile were purchased from Alfa Aesar. Four proteins, bovine serum albumin (BSA), lysozyme (LYZ), cytochrome c (cyt c) from the equine heart, and fibrinogen, were purchased from Sigma-Aldrich. EDOT–OH was purchased from Angene Chemical. All of these chemicals were used without further purification. Besides, the synthesis procedures for EDOT–PO_4_ and EDOT–COOH monomers are described later in the text.

### 2.2 Synthesis of EDOT–PO_4_

Phosphoryl chloride (1.26 mL, 9.0 mmol) was dissolved in diethyl ether (10 mL) in a 25 mL flame-dried round-bottom flask under a N_2_ atmosphere. Triethylamine (1.26 mL, 9.0 mmol) was added to the solution, which was then cooled to −15 °C. EDOT–OH (861 mg, 5.0 mmol) was added to the solution and stirred for 1 h at −15 °C. The reaction was warmed to room temperature and stirred for 1 h. After most of the solvent was evaporated with a rotavapor, the resulting residue was dissolved in DCM (15 mL) and then washed with saturated aqueous NaHCO_3_ solution (3 × 15 mL). The organic layer was dried over anhydrous MgSO_4_, filtered, and the solvent was removed under reduced pressure to give EDOT–PO_4_ monomer as a yellow oil. ^*1*^*H NMR* (400 MHz, CDCl_3_) δ 6.43–6.37 (m, 2H), 4.56–4.45 (m, 3H), 4.28 (d, *J* = 11.7 Hz, 1H), 4.15 (dd, *J* = 11.7, 5.5 Hz, 1H) (Figure S1). *HRMS* (ESI, [M]^−^) for C_7_H_8_O_6_PS calcd 250.9792, found 250.9785.

### 2.3 Synthesis of EDOT–COOH

The EDOT–COOH monomer was synthesized following a previously described method.^35^ In brief, a 100 mL round-bottom flask was equipped with a stir bar, EDOT–OH (861 mg, 5.0 mmol), NaI (150 mg, 1.0 mmol), and NaH (60% suspension in mineral oil, 240 mg, 6.0 mmol). The flask was flushed with nitrogen three times. Dry tetrahydrofuran (THF; 20 mL) was added, and the suspension was stirred for 15 min and cooled in an ice bath. Methyl bromoacetate (0.57 mL, 0.92 g, 6.0 mmol) was added dropwise, and the reaction mixture was stirred for 18 h. Most of the THF was removed with a rotavapor. The crude product was partitioned between water and ethyl acetate, and the aqueous layer was extracted twice with ethyl acetate. The combined organic layers were washed with brine, dried with magnesium sulfate, and evaporated. Finally, the crude product was purified by using a silica gel column (hexane/ethyl acetate, 5:1).

### 2.4 Conducting Polymer Film Preparation

The monomer solutions to form poly(EDOT–PO_4_) or poly(EDOT–COOH) were prepared by dissolving 10 mM EDOT–PO_4_ or 10 mM EDOT–COOH into a solution containing 50 mM dioctyl sodium sulfosuccinate and 100 mM LiClO_4_ in anhydrous acetonitrile. Electropolymerization was then performed using an Autolab PGSTAT128N potentiostat (Metrohm, The Netherlands). A three-electrode setup was employed with QCM chips (QSX 301 Au chip, diameter 14 mm, Biolin Scientific Co., Ltd.) as the working electrode. The QCM chips were first cleaned with piranha solution composed of H_2_SO_4_ and H_2_O_2_ (*v* = 3:1). Platinum served as the counter electrode, and Ag/AgNO_3_ (0.01 M AgNO_3_ and 0.1 M tetrabutylammonium perchlorate in acetonitrile) was used as the reference electrode for the nonaqueous solution. We then applied the potential to the working electrode using cyclic voltammetry to form the PEDOT–PO_4_ and PEDOT–COOH films on the QCM chip at room temperature. The scan rate was set to 0.1 V/s, and the applied voltage for depositing the copolymer films ranged from −0.4 to 1.2 V vs Ag/Ag^+^, which was chosen as the moderate oxidation potential for EDOT–PO_4_ and EDOT–COOH.

### 2.5 QCM-D Measurements

The adsorption and desorption between the surface and ions or proteins were studied using a QCM-D instrument (QSense Explorer and Analyzer System) with a quartz crystal resonator (QSX 301 Au sensor) set at a 5 MHz fundamental resonant frequency. Measurements were carried out in the QFM401 QSense Flow module at a flow rate of 35.0 μL/min, controlled by a tubing pump. Data were recorded as changes in the energy dissipation factor (Δ*D*) and normalized frequencies (Δ*f*_*n*_/*n*) over time. In this study, all frequency and dissipation measurements were taken from the third overtone (*n* = 3) because the first overtone is too sensitive to vibration interference, and the 5th to 13th overtones provide nearly comparable information. Thus, Δ*f* represents Δ*f*_3_/3 for the sake of simplicity. For soft coatings like polymers in a liquid phase, the viscous environment dissipates oscillation energy, and the frequency changes from the QCM-D test correlate positively with the mass of adsorption. Before starting the QCM test, we ensured a stable baseline in the running solutions. Frequency and energy dissipation changes were extracted by subtracting the equilibrium baseline value of the background solutions. Besides, all QCM measurements were performed at 25 °C.

## 3 Result and Discussion

### 3.1 Ion Specificity on Cation Chelation

**Figure 1:**
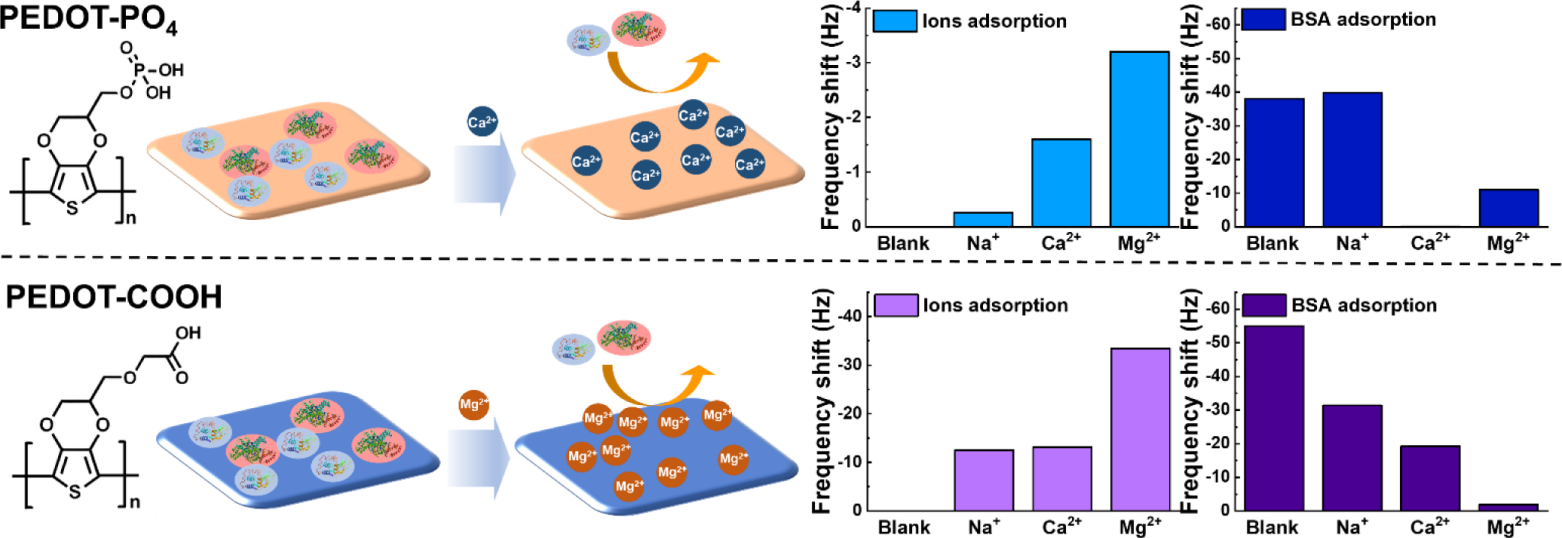
Illustration of how ion chelation inhibits BSA adsorption on PEDOT–PO_4_ and PEDOT–COOH surfaces.

We initially examined the interaction between the ions and the anion-functionalized PEDOT surface. As shown in Figure 1, three ion types were selected: Na^+^ and the divalent ions Ca^2+^ and Mg^2+^, due to their relevance in physiological environments and potential biomedical applications. Using a 10 mM ion solution, we conducted QCM measurements. The results, summarized in a bar plot, indicate that the PEDOT–PO_4_ surface absorbs more divalent cations. While Na^+^ ions are easily rinsed off with deionized (DI) water, divalent ions remain on the surface due to chelation. After rinsing (*t* = 50 min), the frequency shift for Na^+^ was approximately −0.26 Hz, compared to −1.6 Hz for Ca^2+^ and −3.2 Hz for Mg^2+^, suggesting that divalent ions chelate more effectively than monovalent ions on the PEDOT–PO_4_ surface. Next, we tested three types of ions on the PEDOT–COOH surface. Since the adsorption of Mg^2+^ ions with carboxylic groups is significantly higher than that of Na^+^ and Ca^2+^ due to its higher affinity toward carboxyl groups and its stronger bonding with water molecules, we performed QCM measurements using a 1.0 mM MgCl_2_ solution and 10 mM NaCl and CaCl_2_ solutions. The frequency shifts caused by Na^+^ and Ca^2+^ ions are much larger than that of PEDOT–PO_4_, −12.5 and −13.1 Hz, respectively, while the frequency shift for 1.0 mM Mg^2+^ ions is −33.4 Hz. This indicates that Na^+^ and Ca^2+^ ions persist in smaller quantities after DI water rinsing (*t* = 50 min), with Na^+^ exhibiting comparable retention to Ca^2+^ due to a coordination effect with carboxyl groups. In contrast, Mg^2+^ remains on the surface in larger quantities, exhibiting a stronger chelation effect.

### 3.2 Ion Influence on Antifouling Properties

Next, we investigated the antifouling properties of the surface chelated with different ions. As shown in Figure 1, the quantity of BSA binding to the surface without divalent ions is much higher than that of the surface chelated with divalent ions. The frequency shift from BSA adsorption on the PEDOT–PO_4_–Na surface after DI water rinsing (at *t* = 60 min) is about −39.8 Hz, similar to that on the PEDOT–PO_4_ surface without ion chelation. However, the frequency shifts for the PEDOT–PO_4_–Mg and PEDOT–PO_4_–Ca surfaces are much lower. The PEDOT–PO_4_–Mg surface shows a frequency shift of about −11.0 Hz, significantly reducing BSA adsorption. Moreover, the PEDOT–PO_4_–Ca surface exhibits an even lower frequency shift of about −0.11 Hz, demonstrating its outstanding antifouling property. While Mg^2+^ ions appear to chelate more effectively on the PEDOT–PO_4_ surface compared to Ca^2+^ ions, they exhibit weaker antifouling properties against BSA. We attribute this interesting phenomenon to two factors: first, unlike Ca^2+^ ions, which have a similar hydration property as phosphate groups and therefore form stronger bonds with the phosphate groups on the surface, Mg^2+^ ions have a higher water affinity, resulting in weaker bonding with phosphate groups.^36^ This makes Mg^2+^ ions more prone to being washed away by water, reducing their antifouling ability. Additionally, as shown in Figure 1, Mg^2+^ ions exhibit a much higher affinity for carboxyl groups than for phosphate groups. This suggests that Mg^2+^ ions may be displaced due to competition with the carboxyl groups on the protein during the BSA binding test, further weakening their antifouling performance.

Antifouling tests on the PEDOT–COOH surface used 10 mM ion solutions to assess chelation and BSA adsorption. After rinsing with DI water, the frequency shift from BSA adsorption on PEDOT–COOH–Na was −31.3 Hz, while for Ca^2+^ and Mg^2+^ it was much smaller, at −19.2 and −1.9 Hz, respectively. This shows reduced BSA adsorption on PEDOT–COOH–Ca and PEDOT–COOH–Mg, with PEDOT–COOH–Mg exhibiting the best antifouling properties. The chelation of divalent ions on PEDOT–COOH significantly improves antifouling performance compared to monovalent ions. We also measured the contact angle before and after divalent ion chelation. Static contact angles were measured five times by using a contact angle goniometer (Sindatek, Taiwan) at room temperature. As shown in Figure S2, the contact angles of the anionic PEDOT films changed following chelation with divalent metal ions. The contact angle of the EDOT–PO_4_ film decreased significantly from 35.46° to 27.79° after chelation with a 50 mM CaCl_2_ solution, a reduction of 21.63%. These results suggest that Ca^2+^ ion chelation significantly enhances the hydrophilicity of the EDOT–PO_4_ film. Similarly, the contact angle of the EDOT–COOH film was initially 37.74°. After chelation with a 1 mM MgCl_2_ solution, it decreased to 32.89°, representing a reduction of 4.85°. This decrease indicates an increase in surface hydrophilicity, which is likely to contribute to the film’s antifouling properties.

### 3.3 Dissociation Constant for Ion-Surface Chelation

**Figure 2:**
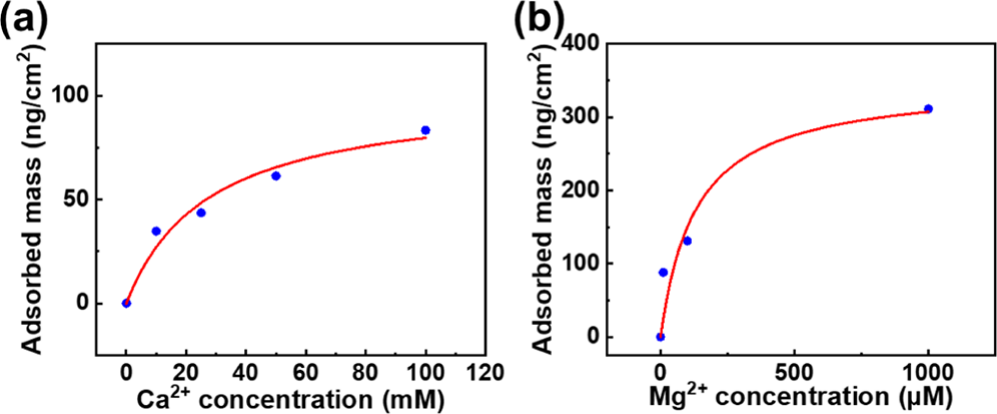
Kinetics of the interaction between divalent cations and the surface. Different concentrations of (a) Ca^2+^ ions were tested on the PEDOT–PO_4_ surface , and (b) Mg^2+^ ions were tested on the PEDOT–COOH surface at a constant flow rate of 35 μL/min.

To further evaluate the antifouling performance of the PEDOT–PO_4_–Ca chelation surface, we calculated the affinity between the surface and Ca^2+^ or Mg^2+^ ions. First, we measured the frequency shift of ion solutions at varying concentrations using a Quartz Crystal Microbalance with Dissipation Monitoring (QCM-D). The frequency shifts were then converted to adsorbed mass changes using the Sauerbrey equation (eq 1). In this equation, Δ*f* represents the resonance frequency shift, Δ*m* denotes the adsorbed mass change on the surface, and *n* represents the number of the odd harmonic (*n* = 3 in this case). The sensitivity constant, *C*, for a 5 MHz crystal is 17.7 ng/(cm^2^·Hz). In general, the Sauerbrey equation is used to explain adsorption behavior in a vacuum or air. However, the Sauerbrey relationship can also describe the behavior of a rigid surface in a liquid when it meets the rigidity criteria, given by Δ*D*/(Δ*f*/*n*) ≪ 0.4 × 10^–6^/Hz,^37,38^ where Δ*D* represents the change in dissipation. After confirming that the surfaces used in this study meet the rigidity criteria, we determined the adsorbed mass of ions at different concentrations and calculated the dissociation constant (*K*_d_) using the Langmuir equation (eq 2), where *Q* represents the adsorbed mass and [M^2+^] denotes the divalent cation concentration in the aqueous solution. The dissociation constant indicates the concentration required to achieve half of the maximum adsorbed mass, with a smaller *K*_d_ reflecting a greater affinity between the surface and the ions. As shown in Figure 2, the dissociation constant between Ca^2+^ and the PEDOT–PO_4_ surface was found to be 27.51 mM, while the dissociation constant between Mg^2+^ and the PEDOT–COOH surface was 131.75 μM.
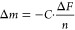
1
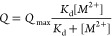
2

### 3.4 Effect of Ion Concentration on Antifouling Properties

**Figure 3:**
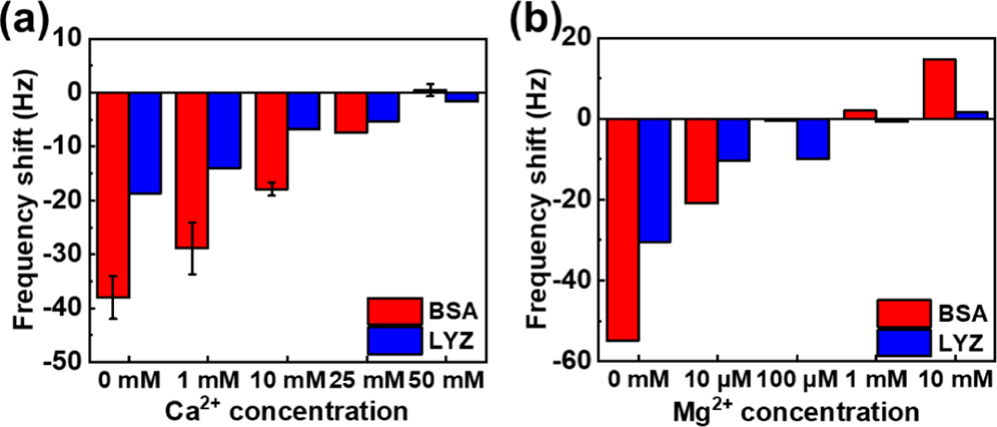
Variation in frequency shift due to BSA and LYZ adsorption on surfaces: (a) chelating different concentrations of Ca^2+^ ions on PEDOT–PO_4_ and (b) chelating different concentrations of Mg^2+^ ions on PEDOT–COOH.

After calculating the dissociation constants between surfaces and cations, we conducted protein binding tests. First, a CaCl_2_ solution (1–50 mM) was flowed over the surface for 25 min, followed by rinsing with DI water. Once the PEDOT–PO_4_–Ca chelation surface stabilized, a 1 mg/mL BSA solution was applied for 25 min and rinsed again. The frequency shift at *t* = 60 min indicated the amount of BSA adsorbed. As shown in Figure 3a, BSA adsorption without Ca^2+^ chelation resulted in a −38.0 Hz shift, which decreased with higher CaCl_2_ concentrations: −28.9 (1 mM), −17.9 (10 mM), −7.4 (25 mM), and −0.46 Hz (50 mM). At a 50 mM concentration, Ca^2+^ ions bind to almost all sites on the surface, demonstrating the best antifouling property toward the BSA protein. Additionally, we investigated the antifouling property of the lysozyme (LYZ) protein. The results were similar to those for BSA, with LYZ adsorption decreasing as the concentration of the CaCl_2_ solution increased. The frequency shift decreased from −18.7 Hz (without Ca^2+^ ion incubation) to −1.7 Hz (after incubation in 50 mM CaCl_2_), showing its antifouling properties in the case of negatively charged BSA and positively charged LYZ.

The antifouling performance of the PEDOT–COOH–Mg surface was also evaluated through protein adhesion tests. To chelate the surface, MgCl_2_ solutions ranging from 10 μM to 10 mM were flowed over the PEDOT–COOH surface for 25 min, followed by DI water rinsing to remove unchelated ions. The chelated PEDOT–COOH–Mg surface was then immersed in a 1 mg/mL BSA solution for another 25 min and rinsed with DI water to complete the antifouling test. Figure 3b shows the frequency shifts resulting from BSA and LYZ adsorption on the PEDOT–COOH–Mg surface after chelation with different concentrations of Mg^2+^ ions. The frequency shift due to BSA adsorption on the surface without Mg^2+^ ion chelation is −55.0 Hz, and that for LYZ is −30.5 Hz. As the Mg^2+^ ion concentration increases, the frequency decreases, indicating less protein binding. For BSA, a 10 μM Mg^2+^ concentration causes a −20 Hz frequency shift, while at 100 μM, the shift is only −0.4 Hz, showing strong resistance to BSA adsorption at low Mg^2+^ levels. At higher concentrations (1 and 10 mM), positive frequency shifts of 1.94 and 14.6 Hz occur, suggesting that both BSA proteins and Mg^2+^ ions leave the surface. This may be due to competition between the carboxyl groups on the surface and those of the proteins for Mg^2+^ binding. In addition, the binding test for positively charged LYZ also shows a decreasing frequency shift when the concentration of Mg^2+^ ions increases. The frequency shift of LYZ adsorption is less than that of BSA due to the electrostatic force of Mg^2+^ ions and LYZ, and the frequency shift decreases to 1.55 Hz at a concentration of 10 mM.

Additionally, to evaluate the stability of the ion-chelated surfaces, we assessed the antifouling properties of PEDOT–PO_4_–Ca, as illustrated in Figure S3a. We conducted a two-step protein binding test by first introducing a 1 mg/mL BSA solution followed by a 1 mg/mL LYZ solution and compared the results with those from PEDOT–PO_4_ without Ca^2+^ chelation. PEDOT–PO_4_ without Ca^2+^ chelation exhibited a frequency shift of −20.1 Hz after LYZ introduction, which increased to −38.6 Hz with BSA adsorption. In contrast, PEDOT–PO_4_–Ca showed only a minimal shift of −0.07 Hz with LYZ and −0.57 Hz after BSA adsorption, indicating stable antifouling behavior. Figure S3b compares the antifouling performance of PEDOT–COOH–Mg and PEDOT–COOH after two consecutive introductions of 1 mg/mL BSA. PEDOT–COOH demonstrated frequency shifts of −39.1 and −68.6 Hz, while PEDOT–COOH–Mg exhibited a shift of 2.7 Hz initially and −26.8 Hz after rinsing. Although not fully reversible, PEDOT–COOH–Mg displayed a significantly reduced nonspecific adsorption.

### 3.5 XPS Analysis

**Figure 4:**
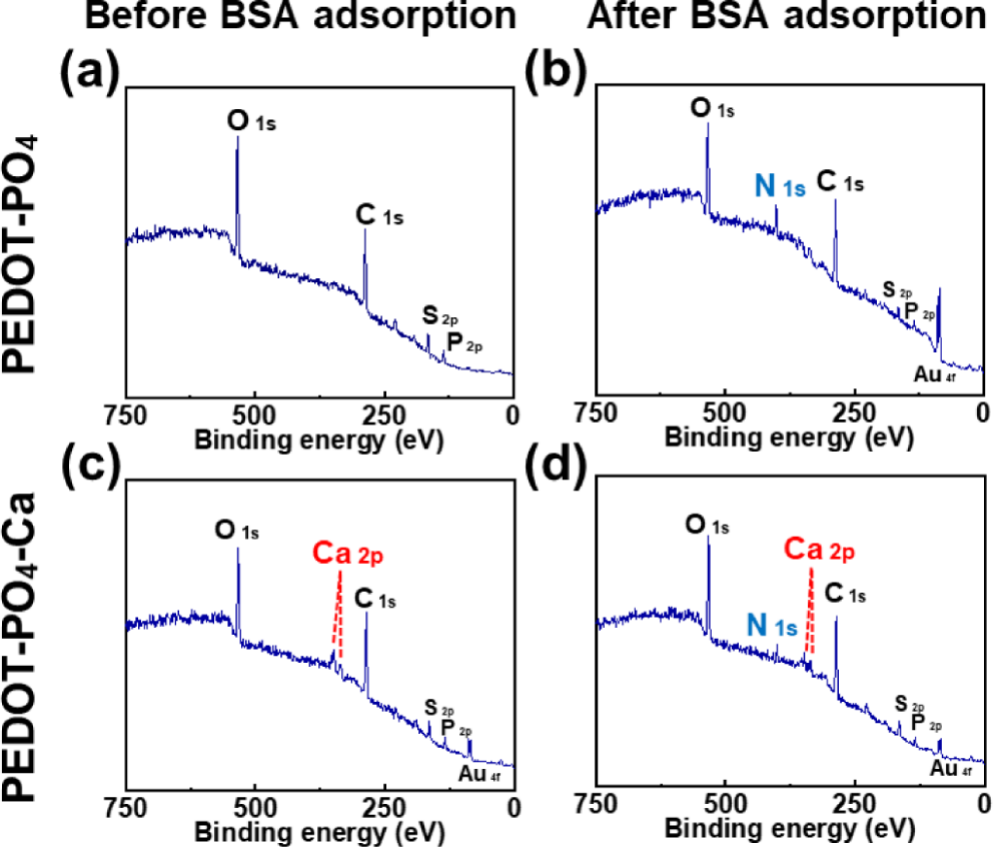
XPS spectrum of (a) PEDOT–PO_4_ surface before the BSA binding test, (b) PEDOT–PO_4_ surface after the BSA binding test, (c) PEDOT–PO_4_–Ca surface before the BSA binding test, and (d) PEDOT–PO_4_–Ca surface after the BSA binding test.

In addition to investigating the antifouling properties using QCM-D, we also examined the surface composition using X-ray photoelectron spectroscopy (XPS) before and after ion chelation and after the BSA adsorption test. Figure 4 presents XPS data for PEDOT–PO_4_ surfaces, showing a peak at 133.3 eV, indicating the presence of phosphorus in the phosphate group. Figure S4a displays two peaks at 284.5 and 286.0 eV in the C 1s range, corresponding to the C–C and C–O/C–S linkages. Figure S4b reveals a Ca 2p peak on the PEDOT–PO_4_–Ca surface, confirming stable chelation between PEDOT–PO_4_ and Ca^2+^ ions. Additionally, fitting the N 1s peak using the Lorentz function showed that after the BSA binding test, the N 1s integral area on the PEDOT–PO_4_ surface was 3198.5, while it decreased to 1714.4 on the PEDOT–PO_4_–Ca surface, aligning with the QCM-D results.

**Figure 5:**
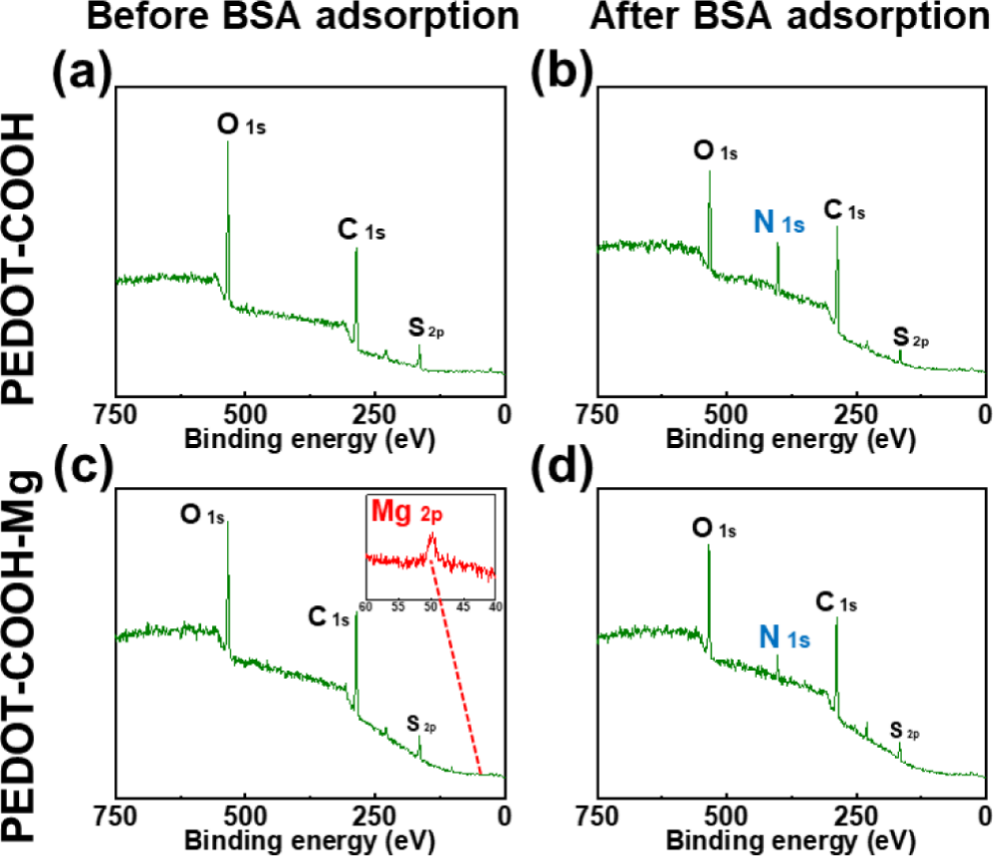
XPS spectrum of (a) PEDOT–COOH surface before the BSA binding test, (b) PEDOT–COOH surface after the BSA binding test, (c) PEDOT–COOH–Mg surface before the BSA binding test, and (d) PEDOT–COOH–Mg surface after the BSA binding test.

Similarly, we analyzed the PEDOT–COOH surfaces by using XPS. Figure 5 shows the XPS results for PEDOT–COOH surfaces, revealing a nitrogen peak after the BSA binding test, particularly on the PEDOT–COOH surface without Mg^2+^ chelation. Figure S6a displays three peaks at 284.2 285.8, and 288.5 eV in the C 1s range, confirming the presence of COOH functional groups and successful PEDOT–COOH film fabrication. In Figure S6b, despite the low sensitivity of Mg in the XPS analysis, the Mg 2p peak is still detectable, indicating chelation between PEDOT–COOH and Mg^2+^. Lastly, Lorentz fitting of the N 1s peak showed an integral area of 5683.1 on PEDOT–COOH after BSA binding, which decreased to 2589.5 on the PEDOT–COOH–Mg surface, highlighting the antifouling properties of the surfaces.

### 3.6 Antifouling Behavior across pH Levels

**Figure 6:**
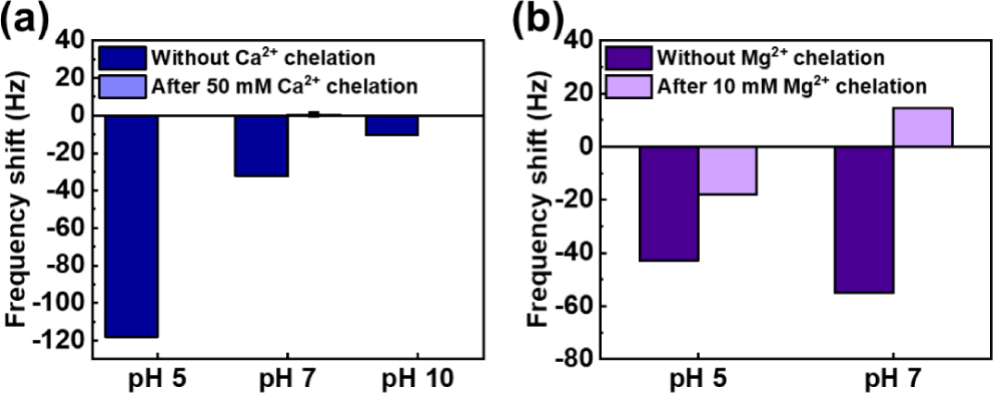
Comparison of frequency shift due to BSA adsorption in different surface pH environments: (a) PEDOT–PO_4_ with or without Ca^2+^ chelation and (b) PEDOT–COOH with or without Mg^2+^ chelation.

Lastly, we conducted a BSA binding test under different pH environments to understand how the interaction between surface charges and proteins influences the adsorption behavior. Figure 6a demonstrates how BSA adsorption on the PEDOT–PO_4_ surface changes with pH. At pH 5, the frequency shift at 60 min is −118.0 Hz, while at pH 7.0 and 10.0, the shifts are significantly lower, at −32.3 and −10.5 Hz, respectively. Without Ca^2+^ ion chelation, the surface becomes more sensitive to pH variations. In acidic conditions, phosphate groups on the surface are protonated, reducing the negative charge, while BSA’s net charge approaches zero near its isoelectric point (pH 4.7), weakening electrostatic repulsion and increasing adsorption. In alkaline conditions, deprotonation of the phosphate groups reduces BSA adsorption. However, when the surface is chelated with 50 mM CaCl_2_, it maintains strong antifouling properties across all pH levels, with frequency shifts remaining below 0.5 Hz. This suggests that Ca^2+^ chelation stabilizes the surface, preventing pH-induced protonation or deprotonation, and minimizing interactions between BSA and the PEDOT–PO_4_–Ca surface.

For the PEDOT–COOH surface, BSA binding tests were also conducted in different environments. Since the *K*_sp_ of Mg(OH)_2_ is 5.6 × 10^–12^ at 25 °C, 10 mM Mg^2+^ ions will form Mg(OH)_2_ precipitate at pH 10, affecting QCM measurements and causing measurement errors. Therefore, tests for PEDOT–COOH–Mg were conducted under pH 5 and 7 environments only. As shown in Figure 6b, for the PEDOT–COOH surface without Mg^2+^ ion chelation, the frequency shifts caused by BSA adsorption are −42.9 and −55.0 Hz under pH 5 and pH 7, respectively. However, under the surface condition with Mg^2+^ ion chelation, the frequency shifts due to BSA adsorption became −18.0 and 14.6 Hz under pH 5 and pH 7. The protonated PEDOT–COOH surface causes a lower frequency shift under pH 5, reducing the chelation effect between COO^–^ and divalent metal ions. Therefore, the PEDOT–COOH surface exhibits better antifouling ability under neutral solutions than under acidic conditions because more COOH groups dissociate, forming more chelation bonds with Mg^2+^ ions on the PEDOT–COOH surface.

We also examined ion chelation under highly acidic conditions, finding that the p*K*_a_ values for carboxylic groups are approximately 3–4, while for phosphate groups, it is around 2 (H_3_PO_4_/H_2_PO_4_). Ion chelation occurred for PEDOT–COOH at pH 3 and PEDOT–PO_4_ at pH 1, as shown in Figure S8. However, chelation decreased due to the protonation of anionic surface groups. For the PEDOT–PO_4_ surface, the frequency shift with a 50 mM CaCl_2_ solution dropped from −15.2 Hz at pH 7 to −4.4 Hz at pH 1. Similarly, for PEDOT–COOH, the frequency shift with a 1 mM MgCl_2_ solution decreased from −33.4 Hz at pH 7 to −9.6 Hz at pH 3. These findings indicate that the protonation of surface functional groups significantly affects the ion chelation.

## 4 Conclusion

In summary, our study uniquely demonstrates the successful synthesis of negatively charged EDOT monomers, EDOT–PO_4_ and EDOT–COOH, and their effective chelation with divalent ions. We observed that PEDOT–PO_4_ films chelated with 50 mM CaCl_2_ solution and PEDOT–COOH films chelated with 1 mM MgCl_2_ solution exhibit outstanding antifouling properties against both BSA and LYZ adsorption. While this study may not directly link to some practical applications, these results underscore the novel approach of utilizing chelation to create zwitterion-like antifouling surfaces, enhancing the understanding of the interaction between charged surfaces and proteins.

## Supporting Information Available

^1^H NMR of EDOT–PO_4_, contact angle variation, two-step stability test, XPS spectrum, pH effect on ion chelation (PDF)

## Abbreviations

PEDOT: poly(3,4-ethylenedioxythiophene)
QCM-D: quartz crystal microbalance with dissipation
BSA: bovine serum albumin
LYZ: lysozyme
Δ*f*: shifts in frequency
Δ*D*: energy dissipation change
XPS: X-ray photoelectron spectroscopy
