# Supplementary material for: Chelation-Induced Zwitterion-like Antifouling Behavior on Anionic Poly(3,4-ethylenedioxythiophene) Surfaces
Source: Langmuir. 2024 Oct 9;40(42):22417–23. doi: 10.1021/acs.langmuir.4c03275 (PMC11500425; doi:10.1021/acs.langmuir.4c03275)
Supplement: Supplementary file 1 — la4c03275_si_001.pdf [file la4c03275_si_001.pdf]

Supporting Information

Chelation-Induced Zwitterion-Like Antifouling Behavior on Anionic Poly(3,4-ethylenedioxythiophene) Surfaces

Tzu-Yu Kao,[†,a] *Ya-Chen Gong,*[†,a] *Cheng-Hsun Huang,*[‡] *Yen-Ku Wu,*[*,‡] *and Shyh-Chyang Luo*[*,†]

[†]Department of Materials Science and Engineering, National Taiwan University, No. 1, Sec. 4, Roosevelt Road, Taipei 10617, Taiwan

[‡]Department of Applied Chemistry, National Yang Ming Chiao Tung University, 1001 University Road, Hsinchu, 30010, Taiwan

NMR spectrum

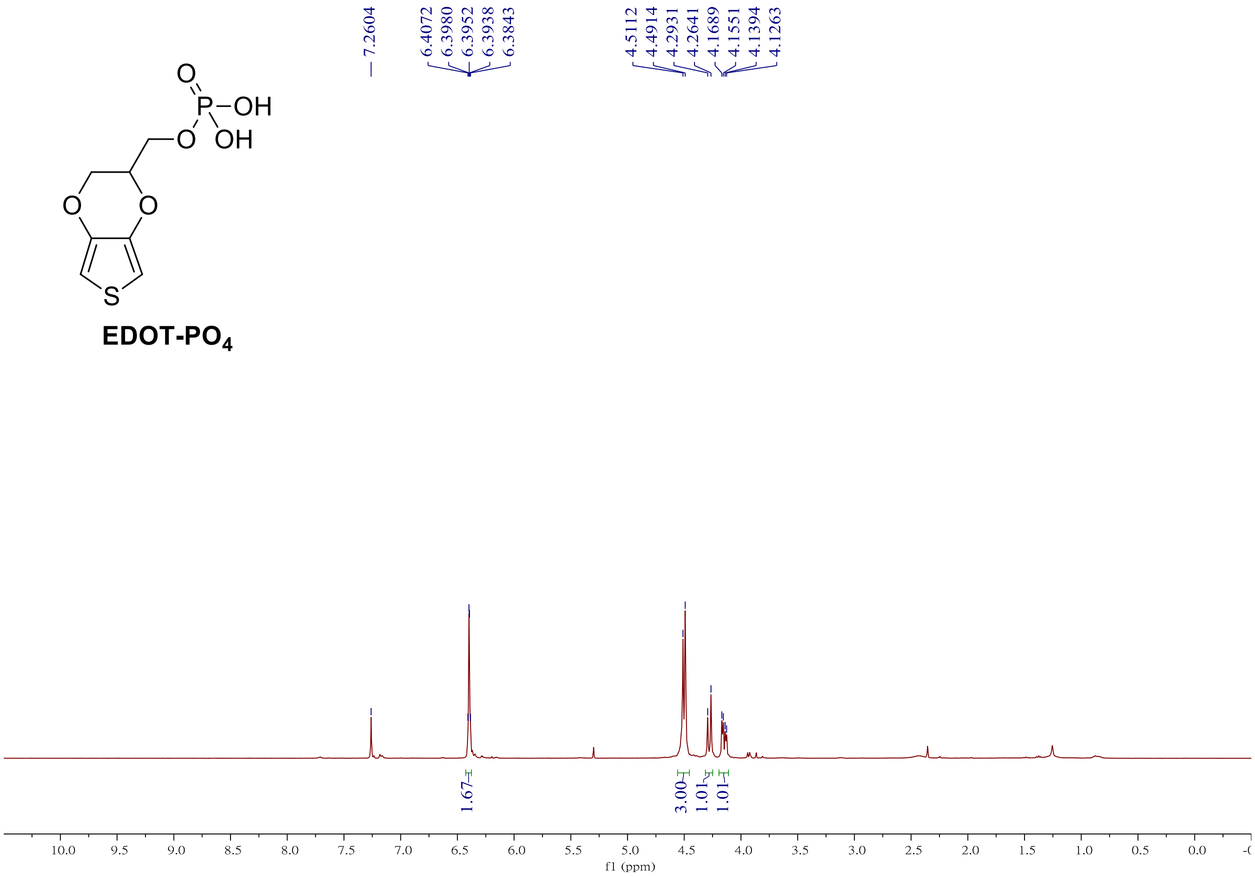

Figure S1. ^{1}H-NMR spectrum in of EDOT-PO_4 in $CDCl_3$.

Contact angle measurement

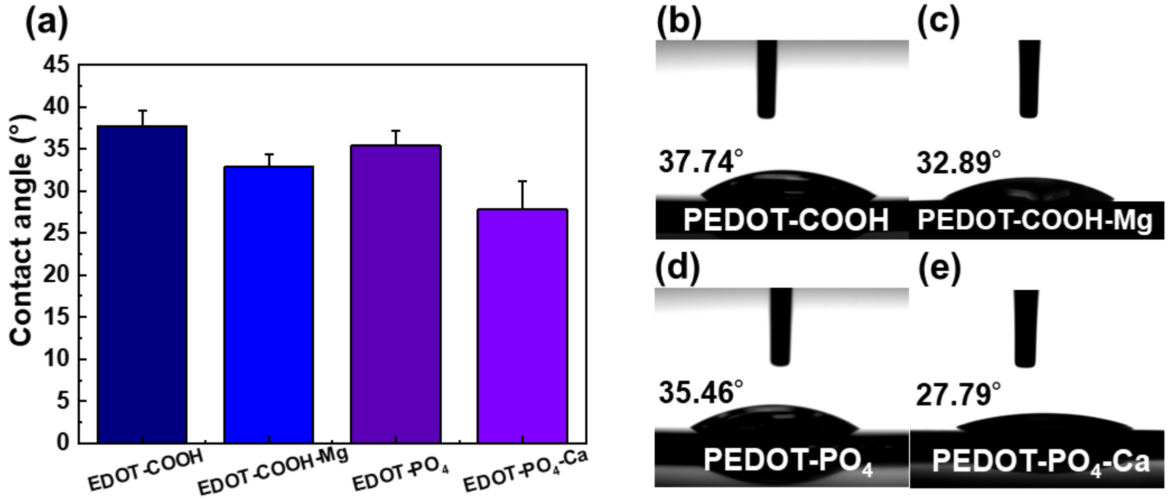

Figure S2. (a) Contact angle variation of anionic PEDOT films before and after chelation with divalent metal ions. Contact angle images of (b) PEDOT-COOH, (c) PEDOT-COOH-Mg, (d) PEDOT-PO_4, and (e) PEDOT-PO_4-Ca.

Two-step stability test

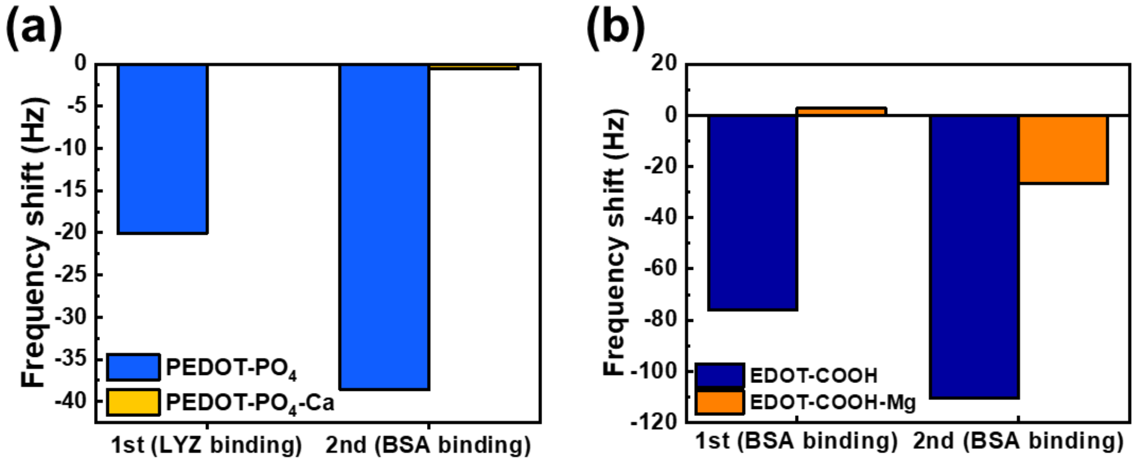

Figure S3. (a) Stability test of the antifouling properties of PEDOT-PO_4 and PEDOT-PO_4-Ca, assessed by introducing BSA followed by LYZ. (b) Stability test of the antifouling properties of PEDOT-COOH and PEDOT-COOH-Mg, evaluated by introducing BSA twice.

XPS spectrum analysis

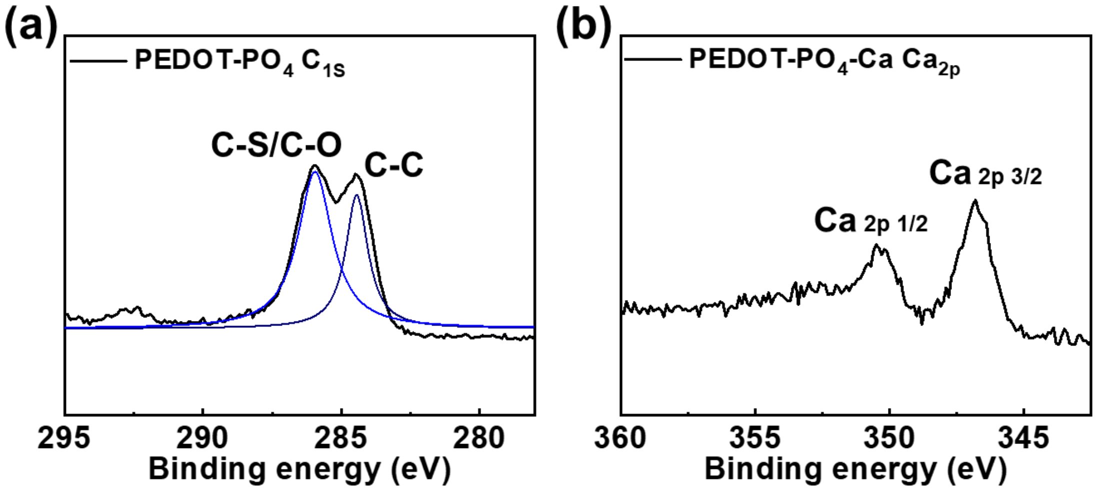

Figure S4. The XPS profile of PEDOT-PO_4-Ca surface: (a) C 1s spectrum; (b) Ca 2p spectrum.

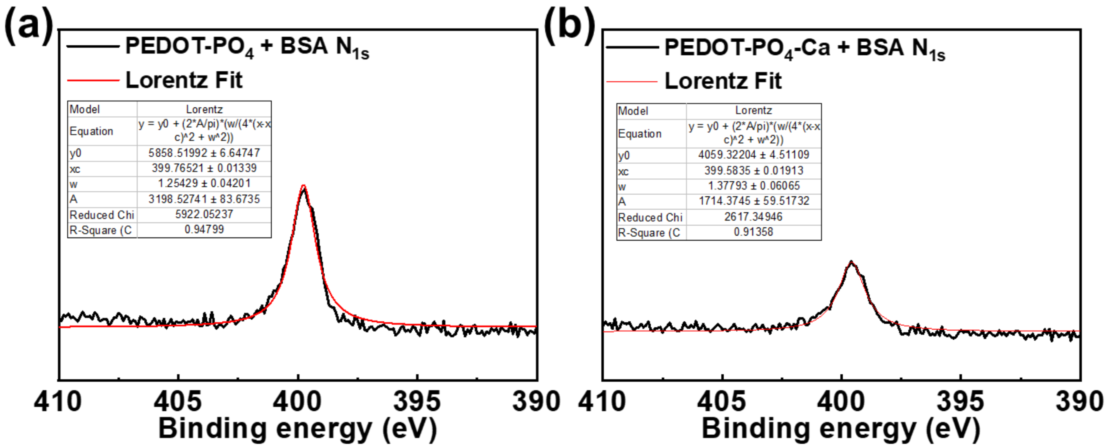

Figure S5. N 1s spectra of (a) PEDOT-PO_4 surface after BSA binding test. (b) PEDOT-PO_4-Ca surface after BSA binding test.

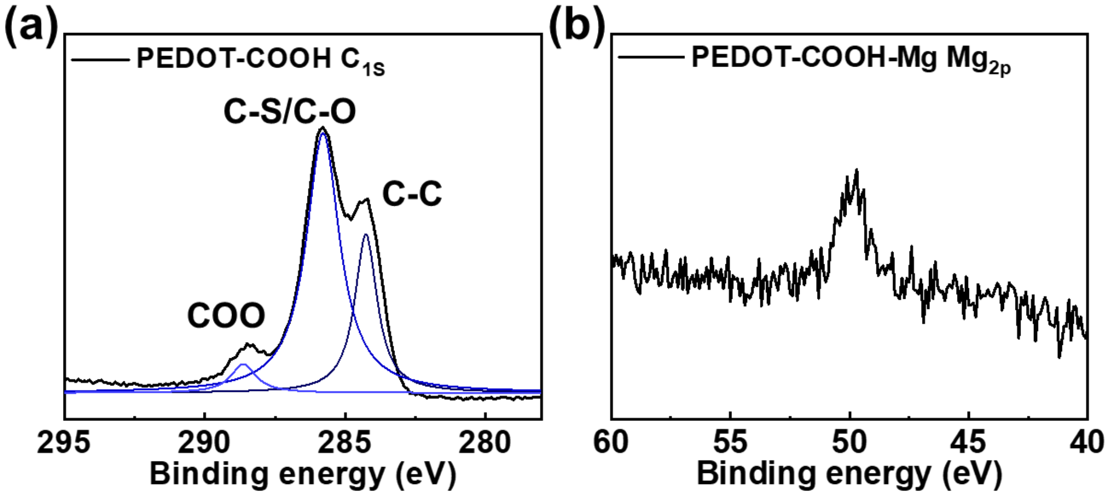

Figure S6. The XPS profile of PEDOT-COOH-Mg surface: (a) C 1s spectrum; (b) Mg 2p spectrum.

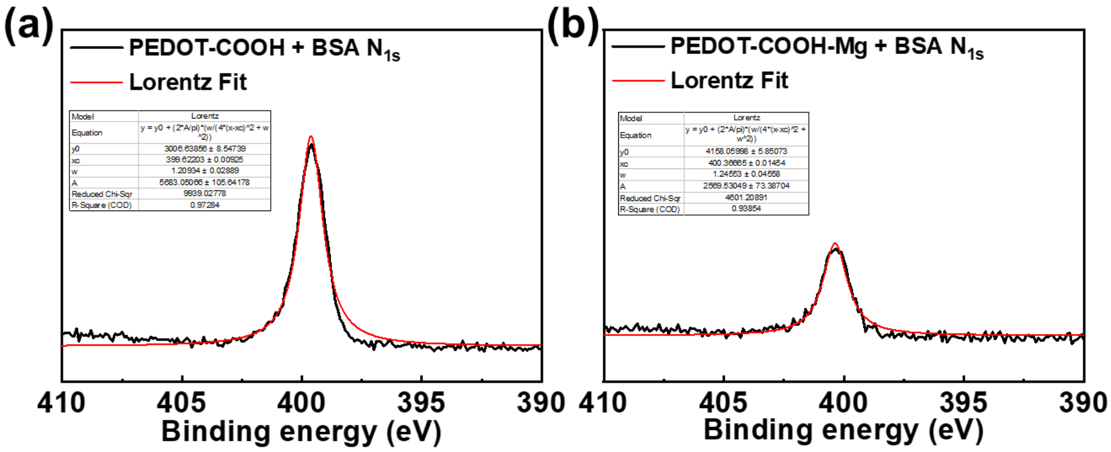

Figure S7. N 1s spectra of (a) PEDOT-COOH surface after BSA binding test. (b) PEDOT-COOH-Mg surface after BSA binding test.

pH effect on ion chelation

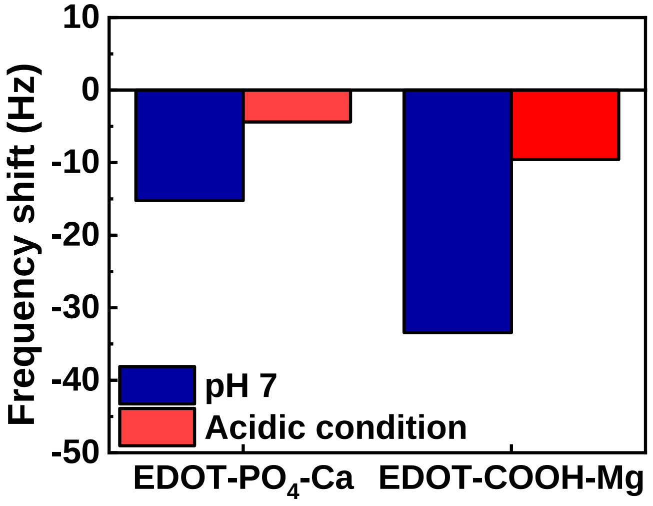

Figure S8. Comparison of ion chelation at different pH values: (a) 50 mM Ca^{2+} on the PEDOT-PO_4 surface at pH 1 and pH 7. (b) 1 mM Mg^{2+} on the PEDOT-COOH surface at pH 3 and pH 7.
